# Supplementary material for: Elevated Levels of Lamin A Promote HR and NHEJ-Mediated Repair Mechanisms in High-Grade Ovarian Serous Carcinoma Cell Line
Source: Cells. 2023 Feb 27;12(5):757. doi: 10.3390/cells12050757 (PMC10001195; doi:10.3390/cells12050757)
Supplement: Supplementary file 1 [file cells-12-00757-s001.zip › cells-2175413-Supplementary materials file S1.pdf]

Elevated levels of lamin A promote HR and NHEJ-mediated repair mechanisms in high-grade ovarian serous carcinoma cell line

Duhita Sengupta[a,b], Asima Mukhopadhyay[c], Kaushik Sengupta[a,b*]
[a]Biophysics and Structural Genomics Division, Saha Institute of Nuclear Physics, Kolkata-700064, West Bengal, India
[b]Homi Bhabha National Institute, Training School Complex, Anushaktinagar, Mumbai- 400094, India
[c] Population Health Sciences Institute, Newcastle University NE2 4BN, United Kingdom

Email: duhita.sengupta@saha.ac.in
kaushik.sengupta@saha.ac.in
asima.mukhopadhyay@newcastle.ac.uk

***Correspondence:**
kaushik.sengupta@saha.ac.in

Supporting Information file S1:

Table S1.

siRNA Sequence:

**siRNA**	**Sequence**
siRNA 1	5'GGUGGUGACGAUCUGGGCU3'
siRNA 2	5'AACUGGACUUCCAGAAGAACAUC3'

Table S2.

Antibodies:

**Name**	**Company**	**Dilution**	**Catalog no**
Lamin A	Sigma-Aldrich	IF: 1:100 ; WB : 1:500	L1293
Lamin B	SantaCruz	IF: 1:50 ; WB : 1:100	sc-6217
γH2AX	EMD-Millipore	IF: 1:50 ; WB : 1:1200	05-636-25UG
Rad51	Abcam	IF: 1:50 ; WB : 1:500	ab63801
BRCA1	EMD-Millipore	IF: 1:50	SAB2702136
BRCA2	Thermo Fischer Scientific	IF: 1:50	234403
Ku70	SantaCruz	IF: 1:50 ; WB : 1:1000	sc-5309
PCNA	SantaCruz	IF: 1:50 ; WB : 1:500	sc-56
βActin	Sigma-Aldrich	WB : 1:1000	A5316
Anti-BrdU	SantaCruz	IF : 1:50	sc-32323
Goat anti-Mouse IgG (H+L) Secondary Antibody, HRP	Thermo Fischer Scientific	WB: 1:400	32430
Goat anti-Rabbit IgG (H+L) Secondary Antibody, HRP	Thermo Fischer Scientific	WB: 1:400	32460

Table S3.

Primer Sequences:

**Primer**	**Sequence**
GAPDH	Forward: 5’- GAAGGTGAAGGTCGGAGTCAAC -3’<br>Reverse: 5’- CAGAGTTAAAAGCAGCCCTGGT -3’
LA	Forward: 5’ -CGGTTCCCACCAAAGTTCA -3’<br>Reverse: 5’-CTCATCCTCGTCGTCCTCAA -3’
LB	Forward: 5’- AAAAGACAACTCTCGTCGCAT- 3’<br>Reverse: 5’ -CCGCTTTCCTCTAGTTGTACG -3’
Rad51	Forward: 5'-TCTCTGGCAGTGATGTCCTGGA-3'<br>Reverse: 5'-TAAAGGGCGGTGGCACTGTCTA-3'
BRCA1	Forward: 5'-CTGAAGACTGCTCAGGGCTATC-3'<br>Reverse: 5'-AGGGTAGCTGTTAGAAGGCTGG-3'
Ku70	Forward: 5'-TGCCACAGGAAGAAGAGTTG-3'<br>Reverse: 5′-CTCTGGAGTTGCCATGATTT-3'
PIF1	Forward: 5’- GGTAAGGTACACAGATTTGAGGC-3’<br>Reverse: 5’-CCCGAGACACCGATAAGTTTT-3’
RIF1	Forward: 5’-TGTTGGAGACTTTGGAAGACC-3’<br>Reverse: 5’-ACTTTGTACAGCCGAGGAAG-3’
BRCA2	Forward: 5’-TTCATGGAGCAGAACTGGTG-3’<br>Reverse: 5’-AGGAAAAGGTCTAGGGTCAGG-3’
FGF2	Forward: 5’-ACCCTCACATCAAGCTACAAC-3’<br>Reverse: 5’-AAAAGAAACACTCATCCGTAACAC-3’
TLR2	Forward: 5’-TGGTAGTTGTGGGTTGAAGC-3’<br>Reverse: 5’- GACAGAGAAGCCTGATTGGAG-3’
BIRC3	Forward: 5’-AATGCTTTTGCTGTGATGGTG-3’<br>Reverse: 5’-GCTTGAACTTGACGGATGAAC-3’
THBS1	Forward: 5’-CTCCCCTATGCTATCACAACG-3’<br>Reverse: 5’-AGGAACTGTGGCATTGGAG-3’
PLK1	Forward: 5’-ACAGTTTCGAGGTGGATGTG-3’<br>Reverse: 5’-GGTTGATGTGCTTGGGAATAC-3’
XRCC2	Forward: 5’-CAGTTGGTGAATGGCGTTG-3’<br>Reverse: 5’-CTACCTTCAAGTCGGGCAAG-3’
POLQ	Forward: 5’-GCCAGGGTTCTCTATGCTTC-3’<br>Reverse: 5’-TCTTCAACTGCTTCCTCTTCC-3’
MCM10	Forward: 5’-AACCAGCCATCAAGTCCATC-3’<br>Reverse: 5’-TGGGCTCTCAACTTCACTTG-3’
BRIP1	Forward: 5’-GCTTAGCCTTACTTTGTTCTGC-3’<br>Reverse: 5’-TTTCACTTACGCCCTCATCTG-3’
TP53	Forward: 5’-GCCATCTACAAGCAGTCACAG-3’<br>Reverse: 5’-TCATCCAAATACTCCACACGC-3’
MYC	Forward: 5’-TTCGGGTAGTGGAAAACCAG-3’<br>Reverse: 5’-AGTAGAAATACGGCTGCACC-3’
TEAD4	Forward: 5’-ATGTTGGAGTTCTCTGCCTTC-3’<br>Reverse: 5’-GGGAATTTGTCATAGATTTGGCG-3’
CTCF	Forward: 5’-GCCATTCAAGTGTTCCATGTG-3’<br>Reverse: 5’-CTCATGTGCCTTTTCAGCTTG-3’

Supplementary Figures:

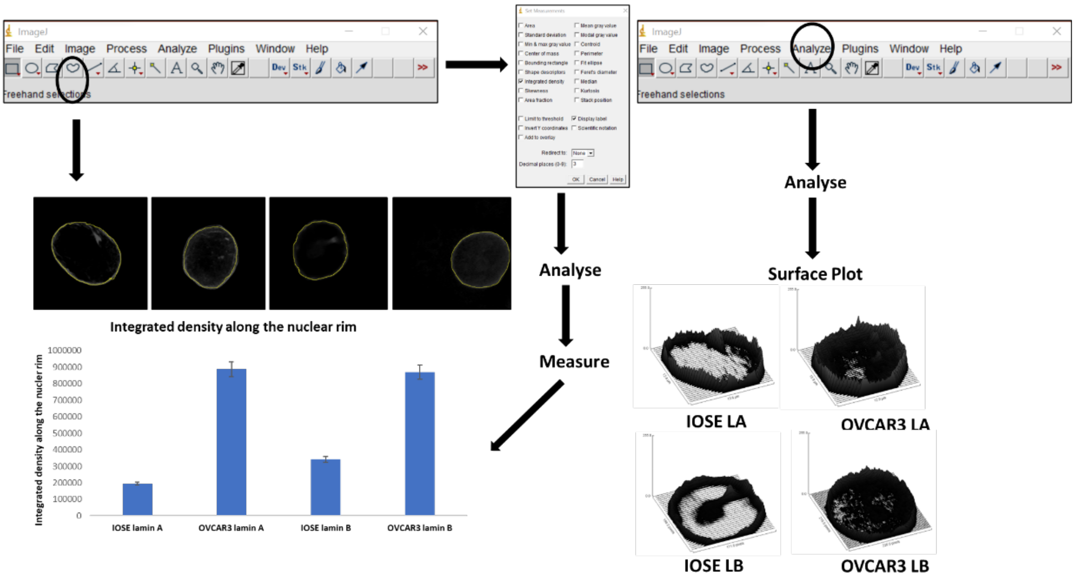

Figure S1: ImageJ work flow for measurement of fluorescence intensities in the nuclear rim. Fluorescence intensities in the nuclear rim have been measured in lamin A and lamin B stained IOSE and OVCAR3 nucleus. 20 nuclei from 10 different fields for each of the samples were used for quantification. Error bar indicates standard error.

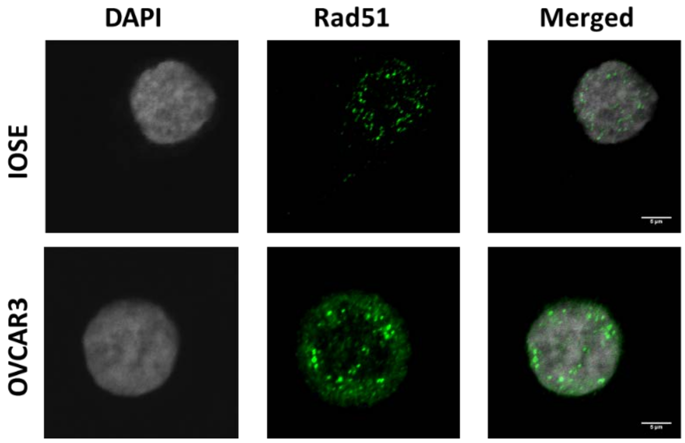

Figure S2. Confocal images of OVCAR3 and IOSE nuclei stained with Rad51. Magnification: 60X. Scale Bar: 5µm

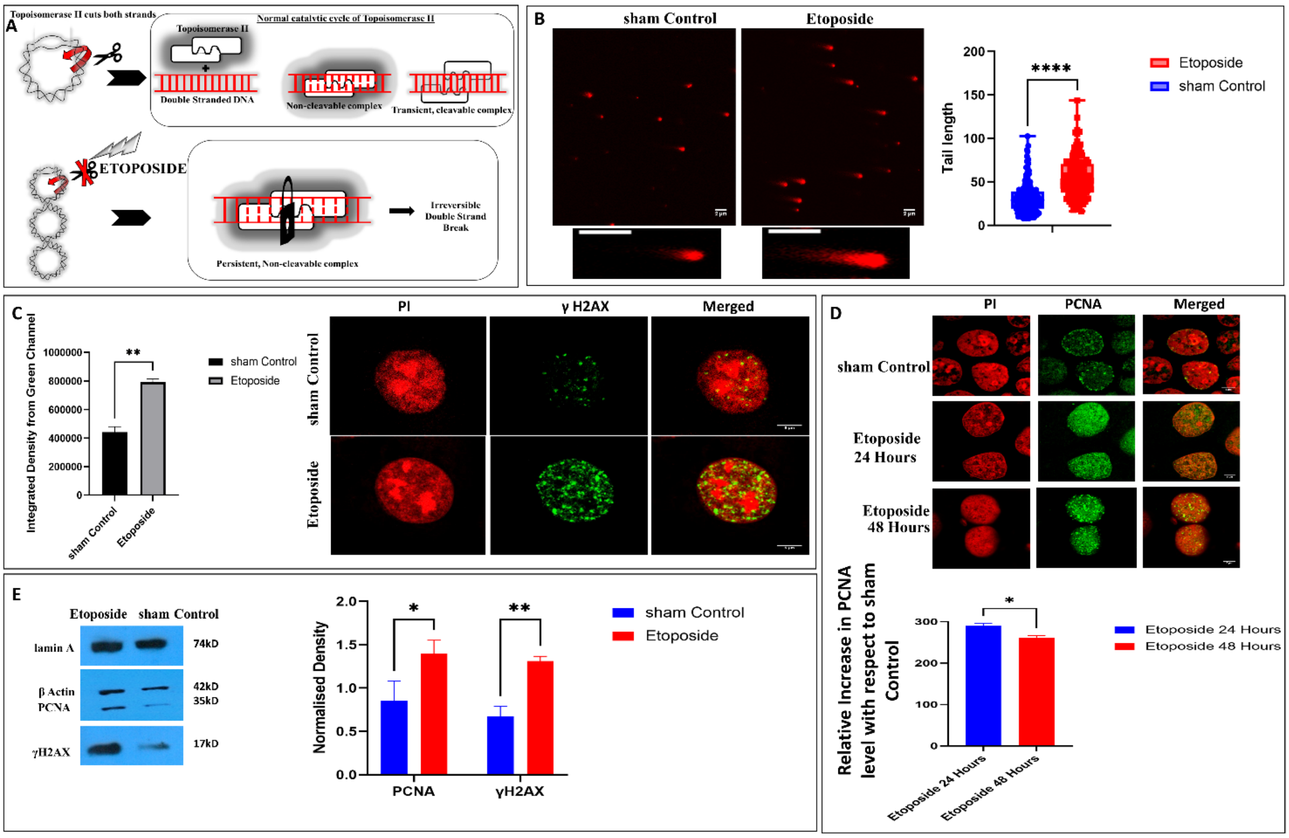

Figure S3: OVCAR3 cells were induced to DNA damage by Etoposide. A. Mode of action of the drug, Etoposide. B. Representative images of neutral comet assays. OVCAR3 cells were treated with Etoposide and investigated by neutral comet assays (Scale bar = 2µm). Tail length and intensities were measured by ImageJ. 15 nuclei from each field were analyzed. 10 such fields were used for quantification from each independent experiment. Error bar indicates standard error of mean. Statistical significance has been analysed by Paired t- test (parametric). p value output is in GP style [0.1234(ns), 0.0332(*),0.0021(**),0.0002(***),<0.0001(****)] C. Confocal micrographs showing the distribution of γH2AX in OVCAR3 cells before and after treatment. Magnification: 60X. Scale Bar; 5µm. The bar diagram shows the integrated density from the green channel (γH2AX). Error bar indicates standard error of mean. Statistical significance has been analysed by Paired t- test (parametric). p value output is in GP style [0.1234(ns), 0.0332(*),0.0021(**),0.0002(***),<0.0001(****)] D. Confocal micrographs showing the distribution of PCNA in OVCAR3 cells before and after treatment for 24 hours and 48 hours. Magnification: 60X. Scale Bar; 5µm. The bar diagram shows the integrated densities from the green channel at 24 hours and 48 hours normalized with green channel (PCNA) intensities of the sham Control. Error bar indicates standard error of mean. Statistical significance has been analysed by Paired t- test (parametric). p value output is in GP style [0.1234(ns), 0.0332(*),0.0021(**),0.0002(***),<0.0001(****)] E. Western Blots showing the level of γH2AX and PCNA. β Actin is used as the loading control. The bar graph shows the normalized density of γH2AX quantified from the blot in treated and untreated OVCAR3 cells. Error bar indicates standard error of mean. Statistical significance has been analysed by multiple t tests (Unpaired, using parametric test, assuming both samples from each row are from populations with the same SD). p value output is in GP style [0.1234(ns), 0.0332(*),0.0021(**),0.0002(***),<0.0001(****)]

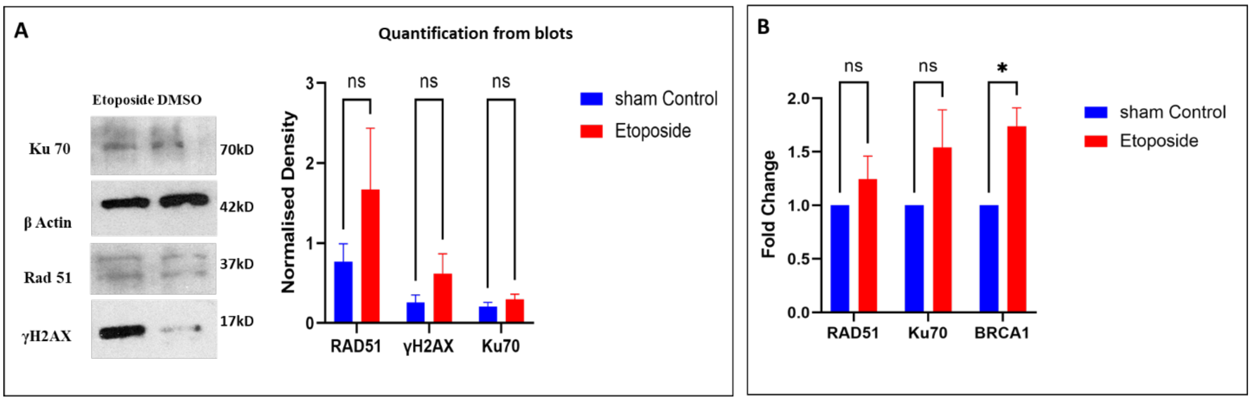

Figure S4: Western Blots showing the level of Ku70, Rad51, and γH2AX in etoposide-treated and untreated OVCAR3 cells. β Actin is used as the loading control. **D.** qPCR showing fold changes of DNA Damage repair proteins in OVCAR3 cells treated with etoposide. Error bar indicates standard error of mean. Statistical significance has been analysed by multiple t tests (Unpaired, using parametric test, assuming both samples from each row are from populations with the same SD). p value output is in GP style [0.1234(ns), 0.0332(*),0.0021(**),0.0002(***),<0.0001(****)]

Sample Name	Overall Alignment Rate
C1 (Mock)	91.30%
C2(Mock)	92.48%
D1(Etoposide)	92.44%
D2(Etoposide)	92.65%

Lane	Project	Sample	Barcode sequence	PF Clusters	% of the lane	% Perfect barcode	% One mismatch barcode	Yield (Mbases)	% PF Clusters	% >= Q30 bases	Mean Quality Score
2	default	C1	TCTGCAAG+AAGGTGAA	8,48,64,895	2.62	95.41	4.59	16,973	100	93.52	35.86
2	default	C2	CAGCGGTA+CCAAGTCA	8,76,81,562	2.71	97.62	2.38	17,536	100	93.57	35.88
2	default	D1	CGCCTTCC+AAGTCCGC	6,20,22,475	1.91	97.77	2.23	12,404	100	93.72	35.91
2	default	D2	CAATAGTC+GTGAGCTG	7,30,12,620	2.25	96.74	3.26	14,603	100	93.77	35.92

Figure S5: Different quality control values and alignment rates of the samples used for RNA Sequencing.

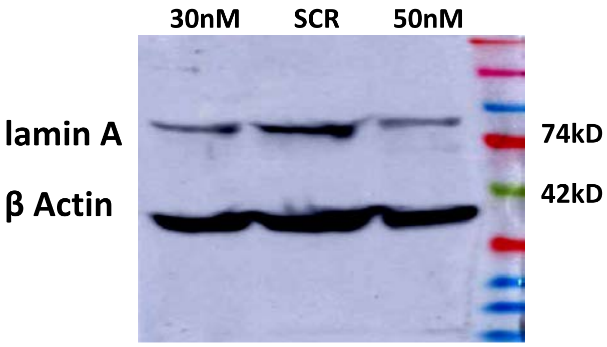

Figure S6: Western blot showing knockdown efficiency of different concentrations of siRNA complexes for lamin A. β Actin is used as loading control.

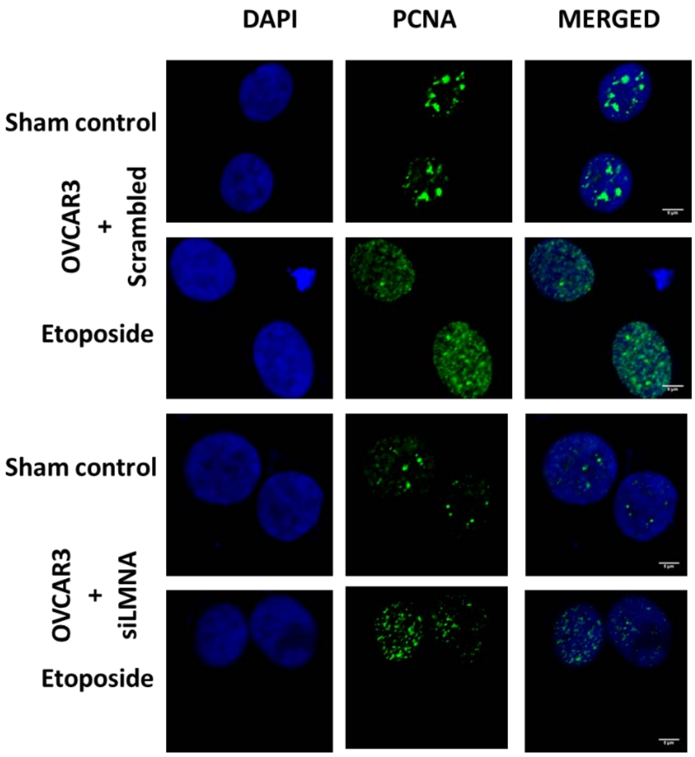

Figure S7: Immunofluorescence images of etoposide treated OVCAR3 nuclei following lamin A knockdown. The first panel shows DAPI staining and the third panel shows the merged images. Magnification: 60X. Scale Bar: 5µm.

Raw images of blots:

1. 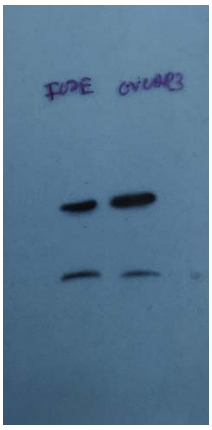

2. 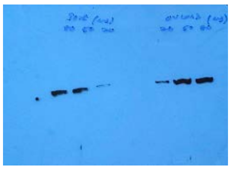

3. 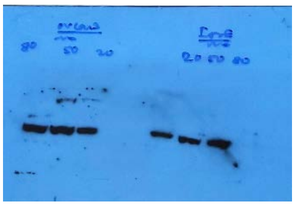

4. 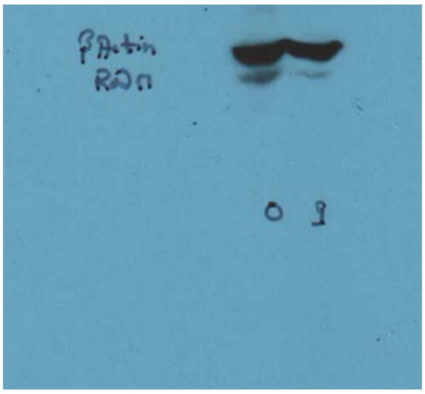

5. 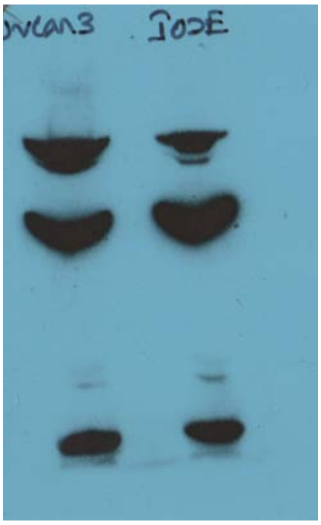

6. 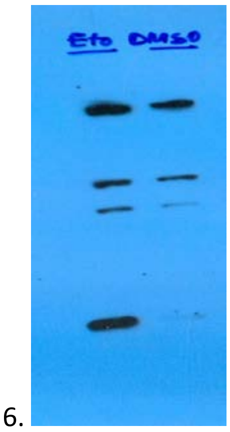

7. 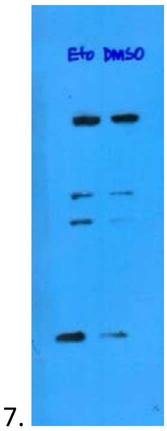

8. 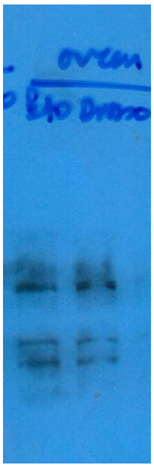

9. 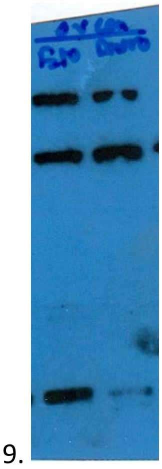

10. 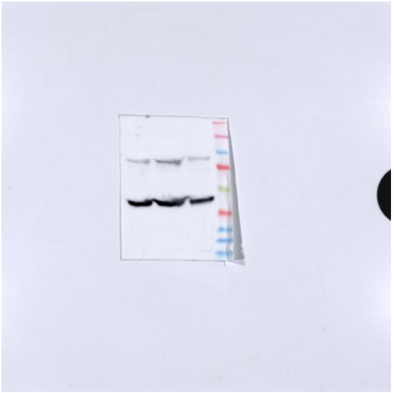

11. 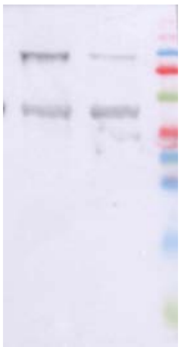

1. lamin A (upper) (pAb)(Rabbit) and β Actin (lower)(mAb)(Mouse)

2. lamin B(pAb)(Goat) (3 no. blot was stripped and probed with lamin B)

3. β Actin(mAb)(Mouse)

4. β Actin(upper))(mAb)(Mouse), Rad 51 (pAb)(Rabbit) (lower)

5. β Actin (upper))(mAb)(Mouse), γH2AX (lower)(mAb)(Mouse) (upper most band denotes lamin A, but that has not been used in this paper. Lamin A blot has been freshly revised)

6. 7th blot in higher exposure time

7. lamin A (upper) (pAb)(Rabbit), β Actin (mAb)(Mouse), PCNA (mAb)(Mouse), γH2AX (lower)(mAb)(Mouse)

8. Ku70 (mouse)(mAb), Rad51 (Rabbit)(pAb), 9 no. blot was stripped and probed with Ku70 and Rad51

9. β Actin (upper))(mAb)(Mouse), γH2AX (lower)(mAb)(Mouse) (upper most band denotes lamin A, but that has not been used in this paper.)

10. lamin A (upper) (pAb)(Rabbit) and β Actin (lower)(mAb)(Mouse)

11. lamin A (upper) (pAb)(Rabbit) and β Actin (lower)(mAb)(Mouse)
